# Inequity in the Distribution of Non-Communicable Disease Multimorbidity in Adults in South Africa: An Analysis of Prevalence and Patterns

**DOI:** 10.3389/ijph.2022.1605072

**Published:** 2022-08-16

**Authors:** R. A. Roomaney, B. van Wyk, A. Cois, V. Pillay-van Wyk

**Affiliations:** ^1^ Burden of Disease Research Unit, South African Medical Research Council, Cape Town, South Africa; ^2^ School of Public Health, University of the Western Cape, Cape Town, South Africa; ^3^ Division of Health Systems and Public Health, Department of Global Health, University of Stellenbosch, Stellenbosch, South Africa

**Keywords:** multimorbidity, disease patterns, latent class analysis, wealth index, South Africa

## Abstract

**Objectives:** The present study examined the prevalence and patterns of non-communicable disease multimorbidity by wealth quintile among adults in South Africa.

**Methods:** The South African National Income Dynamics Study Wave 5 was conducted in 2017 to examine the livelihoods of individuals and households. We analysed data in people aged 15 years and older (N = 27,042), including self-reported diagnosis of diabetes, stroke, heart disease and anthropometric measurements. Logistic regression and latent class analysis were used to analyse factors associated with multimorbidity and common disease patterns.

**Results:** Multimorbidity was present in 2.7% of participants. Multimorbidity was associated with increasing age, belonging to the wealthiest quintile group, increasing body mass index and being a current smoker. Having secondary education was protective against multimorbidity. Three disease classes of multimorbidity were identified: Diabetes and Hypertension; Heart Disease and Hypertension; and Stroke and Hypertension.

**Conclusion:** Urgent reforms are required to improve health systems responsiveness to mitigate inequity in multimorbidity patterns in the adult population of South Africa as a result of income inequality.

## Introduction

South Africa is an upper-middle-income country [1], with one of the highest levels of inequality in the world [2]. The country has a quadruple burden of disease; with mortality trends illustrating that 43% of deaths were due to non-communicable diseases (NCDs), 34% to HIV/AIDS and TB, 14% to other communicable diseases (and perinatal conditions, maternal causes and nutritional deficiencies) and 10% to injuries [3]. It is currently observed that NCDs disproportionately affect people in low and middle-income countries (LMICs), where 85% of premature deaths due to NCDs occur [4].

The observed large increases in NCD burdens in sub-Saharan Africa are driven by the increase in cardiovascular risk factors (i.e., unhealthy diets, physical inactivity, obesity and air pollution) [5]. Multimorbidity is the co-existence of multiple health conditions in an individual [6]. It is reported that one in three people are living with multimorbidity globally [7]. Multimorbidity is associated with increases in healthcare costs and utilization, medication use, hospital admissions, and out of pocket healthcare costs [8]. Individuals with multimorbidity have higher mortality risk [9], a poorer quality of life [10], and complicated medication adherence requirements [11]. The prevalence of NCD multimorbidity in LMICs is already estimated to be as high as 36%. A scoping review highlighted the urgent need for a better understanding of the epidemiology of multimorbidity in LMICs to inform interventions to improve the outcomes of patients with living with multiple diseases [12].

The literature on multimorbidity and socioeconomic status remains divided; possibly due to the way in which multimorbidity is operationalised, as well as varying contexts (e.g., high versus low income countries), and the different ways in which socioeconomic status, wealth and deprivation are measured. A systematic review (focused mainly on high income countries) found increasing levels of deprivation associated with increases in multimorbidity [13]. In cross-sectional studies in high income countries (i.e., South Korea [14] United States, Canada [15], England [15, 16] and Ireland [15]) and China [17] the prevalence of multimorbidity was highest in those with a low socioeconomic status. In contrast, there are reports that multimorbidity could be more prevalent among wealthier people in LMICs [18]. This paper thus reports on the prevalence and patterns of NCD multimorbidity by wealth quintile in a household panel survey of adults in South Africa.

## Methods

### Description of the National Income Dynamics Study Survey Sample and Data Collection

The South African National Income Dynamics Study (NIDS) is the first national household panel study in South Africa that provides information on wealth (livelihoods, how households cope with shocks, poverty, and social capital), sociodemographic (household composition, fertility and mortality, migration, economic activity and education), and health and well-being characteristics [19]. We used data from NIDS Wave 5 conducted in 2017. Health data on diabetes, stroke and heart disease were collected through self-report, and blood pressure and anthropometry measurements were taken at survey administration.

The baseline data collection of the NIDS survey was conducted in 2008 [20], when a two-stage cluster sample design was used to randomly select about 7,300 households across 400 primary sampling units, stratified by district council (a second level administrative division of South Africa’s territory into 52 areas). Data were collected on all members of the selected households, resulting in a total sample size of approximately 28,000 individuals [21]. In the following waves of data collection the same individuals (continuing sample members) were recontacted and interviewed. In addition, all adults belonging to the same household of the continuing sample members at the moment of the interview (temporary sample members) were also interviewed. A top-up sample was recruited in during Wave 5 to compensate for sample attrition and improve representativity of the national population.

This study uses data from Wave 5 of the survey, where 39,400 individuals in 10,800 households were interviewed between February and December 2017 [22]. Additional methodological details from the NIDS Wave 5 survey are available in the Panel User Manual [23].

Ethics approval for data collection for NIDS Wave 1 to 5 was granted by the University of Cape Town’s (UCT) Commerce Faculty Ethics in Research Committee and Faculty of Health Sciences Human Research Ethics [24]. Informed consent in the respondent’s preferred language was obtained for all data collection in the survey. Permission and access to the edited and anonymised dataset (available for public distribution) was obtained from the research data service, DataFirst [24]. The current analysis was approved as part of the lead author’s doctoral studies and received additional ethics clearance by the Biomedical Research Ethics Committee of the University of the Western Cape (BM20/5/8).

### Measures

#### Outcome Variable: Multimorbidity

This study employed a count method for assigning multimorbidity, by counting the number of co-existing disease conditions using a pre-defined list [25]. We created the list of included disease conditions based on recommendations by Holzer et al. [26]. The disease conditions included were based on a predefined list of disease conditions that are frequently included in multimorbidity assessments and disease conditions that are relevant to the South African disease burden. We included self-reported and measured disease conditions. For self-reported diseases, those that could be deemed as “current” were included. For the current analysis study, we included diabetes, heart disease, stroke and hypertension.

For each participant, an index variable was created which added up the number of disease conditions present for each person. If there was missing information for a disease condition, the observation was assumed to have “no disease present.” The Multimorbidity Index was then created by classifying the index variable into those with no disease conditions or one disease condition (i.e., “no multimorbidity”) or those with two or more disease conditions (i.e., “multimorbidity present”).

##### Self-Reported Disease Conditions

Participants were asked if they were ever told by a doctor, nurse or health care professional that they had the disease condition (i.e., diabetes, heart disease and stroke) (Supplementary Table S1). The responses were coded in a binary manner (e.g., disease absent or present).

##### Blood Pressure

For blood pressure measurements, duplicate measurements in the left arm after the participant was seated for at least 5 min [21]. Automated oscillometric devices with standard multi-size cuffs were used to take blood pressure measurements [21]. The average of the replicated readings was considered as the subject’s blood pressure. Replicated measurements of systolic and diastolic blood pressure were assessed for the presence of implausible values (systolic BP < 70 mmHg or >270 mmHg, diastolic BP < 30 mmHg or >150 mmHg), which were set to missing. Hypertension was grouped into “no hypertension present” (systolic BP < 120–139 mmHg & diastolic BP < 80–89 mmHg) or “hypertension present” (systolic BP 140 to ≥160 mmHg & diastolic BP 90 mmHg to ≥100 mmHg) [27]. People on hypertensive medication were included in those that had hypertension.

#### Sociodemographic and Lifestyle Risk Factors

The following variables were investigated as predictor variables of multimorbidity based on the literature [28]: age, sex, locality, educational attainment, employment status, income, asset index, access to medical aid, smoking status and body mass index (BMI).

Employment status was derived using the script available from the NIDS study [23] and based on the International Labour Organization’s definitions of employed, unemployed (strict definition), unemployed (broad definition) and not economically active [23]. Individual income was split into three categories, with the first representing no income and the third representing the highest income.

An asset-based wealth index, based on the 2016 South African Demographic and Health Survey [29, 30], was constructed. The index was created using principal component analyses of questions on:

(a) access to basic services (e.g., household main source of water, type of toilet facility, main source of energy for heating and cooking, and refuse collection),
(b) housing (e.g., number of people living in the dwelling per room, dwelling type, home ownership, material of roof, walls and floors) and
(c) ownership of durable assets (e.g., household has electricity, radio, television, phone, computer, fridge, microwave, gas/electric stove, washing machine, cellphone, bicycle, motorbike, motor vehicle, animal cart and boat).

Each variable was coded as binary (i.e., present or absent), except for the number of people in household per room. Scores were predicted for each household and these scores were then divided into five wealth quintiles—with the 1st representing the lowest quintile (i.e., least wealthy) and the 5th representing the highest quintile (i.e., most wealthy). Each individual in the household was assigned the same quintile.

##### Anthropometry

The NIDS Wave 5 assessed participants based on height and weight measurements taken using a digital scale and stadiometer. The data cleaning followed the procedure used for the BMI risk factor in the second South African Comparative Risk Assessment Study [31]. Implausible values were considered as missing. BMI was calculated using the BMI STATA package and was categorized as follows: underweight (15.0 to <18.5 kg/m^2^), normal weight (18.5 to <25.0 kg/m^2^), overweight (25.0 to <30.0 kg/m^2^), obesity grade 1 (30.0 to <35.0 kg/m^2^), obesity grade 2 (35.0 to <40.0 kg/m^2^), obesity grade 3 (40.0 to <60.0 kg/m^2^) [31].

### Statistical Analysis

Statistical analyses were conducted using STATA 15.0 (Stata Corporation, College Station, TX, United States) software. To account for the complex survey design of the NIDS, including clustering, stratification and unequal selection probability, the STATA survey set (“svy”) of commands were used. Sampling weights calibrated to the represent the South African demographics were used as provided in the original dataset [32]. Weighted data exploration was conducted. Chi-square tests were used to explore bivariate associations between the wealth index quintile and sex, locality, province, educational attainment, employment status, BMI categories and smoking status. Kruskal-Wallis tests were used to test for differences in age and income between the different quintiles in the wealth index. Similarly, Chi-square tests were used to assess single disease conditions and the number of diseases in an individual by wealth index quintile. Multimorbidity status was also described using histograms and box plots against age.

Multivariate logistic regression was employed to assess the relationship between multimorbidity and potential predictors (i.e., age, sex, location, educational attainment, employment, income, wealth index, smoking status and BMI). For the regression, age was categorised according to the United Nations guidelines for age classifications (i.e., 15–24 years, 25–44 years, 45–64 years and 65+ years) [33]. The crude odds ratios were estimated for each predictor variable. The final model included all variables. Model-checking was performed using various statistical tests. The link test [34] was used to determine if there were specification errors. Interaction terms were explored. Pearson residuals, deviance residuals and Pregibon leverage were used to assess influential observations [35]. These tests were done on the unweighted model as they cannot be used on survey weighted data. Crude and adjusted odds ratios were reported with 95% confidence intervals (Cis); *p*-values of less than 0.05 were considered statistically significant.

A latent class analysis (LCA) was performed to explore disease clustering with the four selected disease conditions (i.e., diabetes, stroke, heart disease, hypertension). LCA is a statistical method used to identify sub-groups or classes within populations [36]. The analysis was run using the LCA Stata Plugin as the programme accounts for complex survey design [37, 38]. We conducted the LCA as recommended by Weller et al. [36] For example, to identify latent classes, a one-class model was estimated and then additional classes were added to compare the relative fit of models [36]. The relative fit of models were compared using a series of information indices, namely, the Bayesian information criterion (BIC) [39], the adjusted BIC (aBIC) [40], and the Akaike Information Criterion (AIC) [41]. Lower values of these information indices indicated a better fit [42]. After selecting the model with the best fit, individuals were assigned to the class with the highest posterior probability.

## Results

### Sample Description

The sample consisted of 27,042 participants, with more females (56.9%, *n* = 15,362) than males (Table 1). The median age of the sample was 33 years (IQR: 23–51). In terms of population group, most of the sample were Black African (77.9%), followed by Coloured (13.5%), White (6.5%) and Asian (2.1%). Missing data is reported in Supplementary Table S2.

**TABLE 1:** Description of sample by wealth quintiles (South Africa, 2017, unweighted).

Variable	Total (%, n) N= 27,042	Wealth quintiles (%, n)					*p*-value^a^
		Q1/Least wealthy *n* = 4,772	Q2 *n* = 4,496	Q3 *n* = 4,470	Q4 *n* = 4,541	Q5/Most wealthy *n* = 4,264	
Age (Median and interquartile range in years)^b^	33 (23–51)	32 (21–50)	31 (22–47)	32 (23–46)	33 (23–49)	39 (26–56)	**<0.001**
Sex							**<0.001**
Male	43.2 (11,659)	40.4 (1,926)	43.6 (1,959)	43.3 (1,936)	45.1 (2,050)	45.0 (1,914)	
Female	56.9 (15,362)	59.6 (2,842)	56.4 (2,534)	56.7 (2,531)	54.9 (2,491)	55.0 (2,343)	
Locality							**<0.001**
Rural	44.4 (11,992)	83.9 (4,003)	68.8 (3,093)	39.3 (1758)	21.1 (957)	10.5 (449)	
Urban	55.7 (15,050)	16.1 (769)	31.2 (1,403)	60.7 (2,712)	78.9 (3,584)	89.5 (3,815)	
Province							**<0.001**
Western Cape	11.5 (3,099)	2.1 (99)	6.1 (273)	10.4 (463)	18.9 (857)	22.8 (970)	
Eastern Cape	11.1 (3,012)	16.4 (781)	11.2 (505)	9.9 (441)	10.3 (469)	7.5 (319)	
Northern Cape	7.2 (1,936)	4.7 (226)	7.5 (338)	8.4 (377)	10.5 (476)	6.1 (262)	
Free State	5.5 (1,493)	1.2 (57)	3.6 (161)	9.2 (410)	9.0 (408)	4.8 (206)	
KwaZulu-Natal	28.6 (7,740)	47.1 (2,246)	38.3 (1723)	24.4 (1,091)	15.3 (696)	15.2 (648)	
North West	6.1 (1,640)	3.8 (182)	7.7 (346)	8.1 (361)	6.7 (306)	3.9 (164)	
Gauteng	14.6 (3,960)	5.1 (243)	5.8 (262)	14.3 (637)	17.1 (777)	27.7 (1,182)	
Mpumalanga	7.2 (1,954)	6.2 (294)	8.8 (396)	8.4 (375)	7.6 (345)	6.3 (268)	
Limpopo	8.2 (2,208)	13.5 (644)	10.9 (492)	7.1 (315)	4.6 (207)	5.8 (245)	
Education level							**<0.001**
Primary or less	23.6 (6,320)	39.2 (1866)	27.7 (1,237)	23.2 (1,033)	18.5 (834)	9.2 (388)	
Secondary complete	63.2 (16,952)	57.7 (2,742)	66.2 (2,959)	67.8 (3,015)	67.7 (3,057)	59.2 (2,489)	
Tertiary	13.2 (3,551)	3.1 (147)	6.1 (274)	9.0 (398)	13.9 (628)	31.6 (1,328)	
Employed	33.9 (9,157)	26.4 (1,258)	31.8 (1,429)	37.0 (1,655)	40.4 (1,833)	45.5 (1,939)	**<0.001**
Individual Monthly Income (Median & Interquartile range in ZAR)^c^	0 (0–2000)	0 (0–200)	0 (0–1,100)	0 (0–2,100)	0 (0–3,000)	0 (0–7,706)	**<0.001**
Private health insurance	11.3 (26 90)	0.8 (36)	2.2 (94)	5.6 (233)	11.5 (475)	37.3 (1,415)	**<0.001**
Body Mass Index							**<0.001**
Underweight	8.0 (1,870)	8.8 (388)	8.8 (366)	9.6 (390)	7.6 (308)	5.6 (202)	
Normal weight	42.2 (9,821)	50.0 (2,202)	47.8 (1978)	42.4 (1721)	38.4 (1,565)	32.8 (1,193)	
Overweight	22.8 (5,306)	20.4 (899)	20.7 (857)	22.3 (906)	23.6 (963)	26.9 (977)	
Obesity grade 1	14.6 (3,397)	12.6 (553)	13.1 (544)	13.3 (540)	15.7 (641)	18.3 (664)	
Obesity grade 2	7.4 (1,733)	4.8 (213)	6.1 (254)	7.4 (299)	8.4 (344)	9.6 (350)	
Obesity grade 3	5.0 (1,169)	3.3 (146)	3.5 (143)	5.1 (208)	6.3 (256)	6.9 (252)	
Current smoker	17.7 (4,225)	14.9 (667)	16.6 (702)	19.4 (806)	22.1 (916)	18.3 (695)	**<0.001**

a Chi-square tests used for all variables other than age and income where the Kruskal-Wallist test used.

b Age in years.

c Income in South African Rands (1 US Dollar = 14.5 ZAR on 30 March 2022, https://www.x-rates.com/table/?from=ZAR&amount=1).

Bold values represent p < 0.05 i.e., significant.

Note: There were 4,499 observations with missing wealth quintile information.

Just more than half of the sample lived in an urban location (55.7%). Place of residence varied significantly by wealth quintile, with a high proportion of wealth Quintile 1 living in rural areas (83.9%) and a large proportion of Quintile 5 living in urban areas (89.5%).

Approximately 63% of participants completed secondary education, and tertiary educational attainment was highest in wealth Quintile 5 group. Only a third of the sample was employed and income levels varied by wealth quintiles. Income was heavily skewed to the left with median income being R0 in all quintiles (overall interquartile range: R0–R2000). Approximately 11% of the sample had access to private medical aid, and significantly higher in wealth Quintile 5.

About 42% of the sample had a normal BMI, with females significantly more likely to fall outside the normal BMI range (*p* < 0.001) (Supplementary Table S3). Levels of obesity was highest in wealth Quintile 5. Current smoking status varied by quintiles and peaked in the wealth Quintile 4 group.

### Disease Prevalence in the Population

**FIGURE 1:**
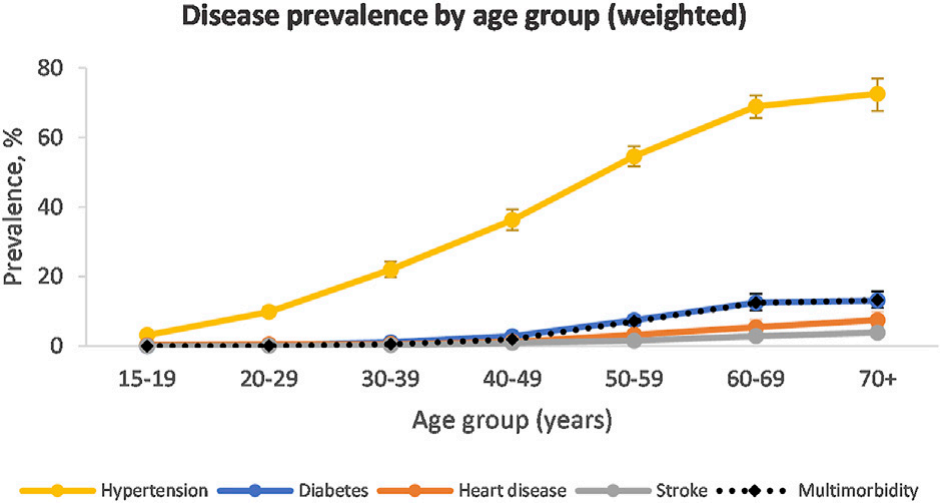
Disease prevalence by age group (South Africa, 2017, weighted).

When taking population weighting into account, hypertension was the most prevalent disease condition in the population (27.8%, 95% CI: 26.7–29.0) (Figure 1). This was followed by diabetes (2.9%, 95% CI: 2.6–3.3), heart disease (1.6%, 95% CI: 1.3–1.8) and stroke (0.8%, 95% CI: 0.6–1.0). The prevalence of hypertension and diabetes rose with increasing wealth quintiles (Supplementary Table S4). The prevalence of heart disease was similar in wealth Quintiles 1–3 but increased in Quintile 4 and 5. The prevalence of stroke did not appear influenced by wealth quintile.

The prevalence of hypertension was similar among males and females (Supplementary Table S4). Hypertension prevalence increased with increasing age and peaked in the 70+ year age group at 70.6% in males and 73.6% in females (Supplementary Figure S1). Diabetes, heart disease and stroke started increasing in the 40–49-year age group and peaked in the 70+ year age group.

The majority of the population had none of the included diseases (74.5%, 95% CI: 73.5–75.4) (Table 2). A further 22.8% (95% CI: 21.8–23.8) had one disease condition. Multimorbidity was present in 2.7% (95% CI: 2.4–3.1) of the population, with it being more prevalent among wealth Quintile 5 compared to the other quintiles. Multimorbidity was also more prevalent among females compared to males (Supplementary Table S6). The prevalence of multimorbidity was low in younger age groups and peaked at 13.2% among the 70+ age group (Supplementary Figure S1).

**TABLE 2:** Number of disease conditions in individuals by wealth index (South Africa, 2017, weighted).

Number of disease conditions	Weighted prevalence (%, 95% CI)					
	Total	Wealth quintiles				
		Q1 (least wealthy)	Q2	Q3	Q4	Q5 (most wealthy)
No diseases	74.5 (73.5–75.4)	77.3 (75.3–79.2)	75.4 (73.3–77.4)	75.0 (72.7–77.1)	71.4 (69.0–73.7)	68.2 (65.9–70.5)
1 disease	22.8 (21.8–23.8)	21.1 (19.2–23.1)	22.5 (20.5–24.5)	23.1 (20.9–25.4)	26.0 (23.6–28.4)	26.9 (24.8–29.0)
2 diseases	2.3 (2.1–2.6)	1.4 (1.1–1.8)	1.6 (1.3–2.1)	1.6 (1.3–2.1)	2.3 (1.7–2.9)	4.2 (3.4–5.2)
3 + diseases	0.4 (0.3–0.5)	0.2 (0.1–0.5)	0.5 (0.2–1.0)	0.3 (0.2–0.5)	0.3 (0.2–0.6)	0.7 (0.4–1.0)
Multimorbidity (≥ 2 diseases)	2.7 (2.4–3.1)	1.6 (1.3–2.1)	2.1 (1.6–2.7)	1.9 (1.5–2.4)	2.6 (2.0–3.4)	4.9 (4.0–6.0)

### Factors Associated With Multimorbidity

Multimorbidity was strongly and significantly associated with age, with the odds of having multimorbidity increasing rapidly among older age groups (Table 3). In the crude analysis, females had higher odds of multimorbidity compared to males, but this did not remain significant in the adjusted model. Locality was not associated with multimorbidity.

**TABLE 3:** Factors associated with multimorbidity (crude and adjusted Odds Ratios, South Africa, 2017).

Variable	Unadjusted odds ratios (95% CI)	Adjusted odds ratios (95%CI)
Age category (Reference category: 15–24 years)		
25–44 years	12.4 (4.8–32.3)*	10.0 (3.4–29.5)*
45–64 years	114.1 (45.8–284.3)*	60.6 (21.5–170.9)*
65+ years	289.2 (114.4–730.8)*	126.7 (44.1–363.7)*
Female (Reference: Male)	2.0 (1.6–2.5)*	1.1 (0.8–1.5)
Urban location (Reference: Rural)	1.2 (1.0–1.5)	1.1 (0.8–1.6)
Education (Reference: Primary)		
Secondary	0.3 (0.2–0.3)*	0.7 (0.5–0.9)*
Tertiary	0.4 (0.3–0.6)*	0.7 (0.4–1.2)
Employed (Reference: Unemployed)	0.5 (0.4–0.7)*	0.7 (0.3–1.7)
Asset Index (Reference: Quintile 1)		
Quintile 2	1.3 (0.9–1.8)	1.5 (1.0–2.2)
Quintile 3	1.2 (0.8–1.6)	1.4 (1.0–2.1)
Quintile 4	1.6 (1.1–2.3)*	1.7 (1.0–2.8)
Quintile 5 (Most wealthy)	3.1 (2.2–4.2)*	2.4 (1.5–3.7)*
Individual Income (Lowest)		
Group 2 (Medium)	0.4 (0.3–0.6)*	0.6 (0.2–1.7)
Group 3 (Highest)	0.5 (0.4–0.7)*	0.6 (0.3–1.7)
Medical aid	2.2 (1.7–2.9)*	1.4 (1.0–2.1)
Current smoker (Reference: No current smoking)	0.7 (0.5–1.0)	1.6 (1.1–2.4)*
BMI categories (Reference: Normal BMI)		
Underweight	0.8 (0.4–1.6)	0.6 (0.3–1.3)
Overweight	3.4 (2.5–4.7)*	2.6 (1.8–3.8)*
Obesity grade 1	5.6 (3.9–8.0)*	3.4 (2.2–5.1)*
Obesity grade 2	6.7 (4.5–9.9)*	3.8 (2.5–5.9)*
Obesity grade 3	6.6 (4.5–9.6)*	3.7 (2.3–5.9)*

*The *p-*value was significant (*p* < 0.05)

Having secondary education was associated with reduced odds of multimorbidity, compared to those with only primary school education (OR: 0.7, 95% CI: 0.5–0.9). While a similar pattern was observed in those with tertiary education, it was not significant in the adjusted analysis—possibly due to a lack of statistical power. People in Quintile 5 (most wealthy) had 2.4 times the odds of having multimorbidity compared to those in Quintile 1 (least wealthy) (95% CI: 1.5–3.7). In the adjusted analysis, there was no significant association between multimorbidity and income, having medical aid or being employment.

When compared to those with normal BMIs, those that were overweight had more than double the odds of multimorbidity (OR: 2.6, 95% CI: 1.8–3.8), those with obesity grade 1 had three times the odds of multimorbidity (OR: 3.4, 95% CI: 2.2–5.1) and those with obesity grade 2 and 3 had almost four times the greater odds of multimorbidity (OR: 3.8, 95% CI: 2.5–5.9 and OR: 3.7, 95% CI: 2.3–5.9, respectively). Smokers were more likely to have multimorbidity than non-smokers (OR:1.6, 95% CI:1.1–2.4).

### Multimorbid Population Analysis

Only the multimorbid sample was included in the subsequent analysis (*n* = 971). The unweighted mean age of people with multimorbidity was 60 years (58.9–61.5 years). Also, 67.7% were female. In terms of included disease conditions (weighted), almost all the multimorbid population had hypertension (98.9%, 95% CI: 97.6–99.5), followed by diabetes (68.2%, 95% CI: 63.0–72.9), heart disease (37.5%, 95% CI: 32.4–43.0) and stroke (16.1%, 95% CI: 13.2–19.4) (Supplementary Table S7). The disease prevalence was similar among the sexes, except for stroke which was more prevalent in males (20.1%, 95% CI: 14.4–27.4) compared to females (14.1%, 95% CI: 11.0–18.0).

**FIGURE 2:**
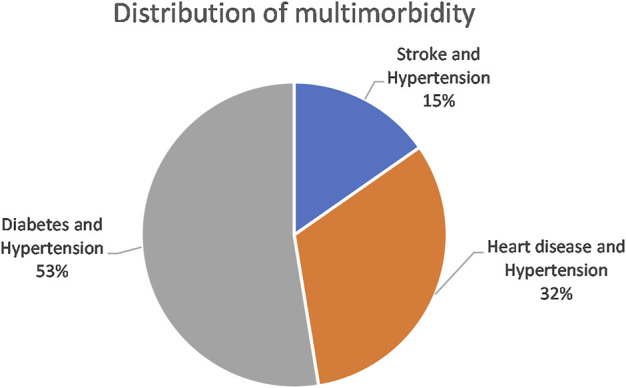
Distribution of latent classes in the multimorbid population (South Africa, 2017, weighted).

Supplementary Table S8 shows a comparison of fit statistics for models with different numbers of classes, ranging from two to five classes. The BIC, adjusted-BIC and AIC were minimal for a three-class model. A four-class model produced a slightly lower AIC but since the AIC tends to prefer over-complicate models, the three-class model was chosen. Classes were named based on the diseases with the highest prevalence in that class. The model identified the following membership, latent classes, from largest to smallest: “Diabetes and Hypertension” (52.6%), “Heart disease and Hypertension” (32.1%), and “Stroke and Hypertension” (15.3%) (Figure 2). A high probability of hypertension was common among all the disease classes. Standard errors are available in Supplementary Table S9.

Table 4 shows the item response probabilities for each disease condition by latent class. The largest class (Diabetes and Hypertension) was characterised by 100% certainty of having both diabetes and hypertension. The second-largest class (Heart disease and Hypertension) was typified by a 99.9% probability of heart disease and a 98.4% probability of hypertension. The smallest class (Stroke and Hypertension) was characterised by very high probabilities of stroke (99.7%) and hypertension (95.6%).

**TABLE 4:** Disease probabilities within classes for the 3-class latent class analysis model (South Africa, 2017).

Class	Disease probabilities (standard errors)			
	Hypertension	Diabetes	Heart disease	Stroke
Class 1: Stroke & Hypertension	**0.956 (0.023)**	0.240 (0.080)	0.298 (0.084)	**0.997 (0.001)**
Class 2: Heart disease & Hypertension	**0.984 (0.007)**	0.335 (0.046)	**0.999 (0.000)**	0.004 (0.005)
Class 3: Diabetes & Hypertension	**1.000 (0.000)**	**1.000 (0.000)**	0.007 (0.003)	0.008 (0.021)

Bold text indicates a high probability of that disease condition within a class.

## Discussion

Our results support the well-established notion of a positive association between increase in age and NCD multimorbidity; as shown in a recent systematic review of multimorbidity in 22 out of 25 studies in LMICs [43]. South Africa’s population is ageing—the proportion of elderly persons (60 years and older) increased from 7.6% in 2002 to 9.1% in 2020 [44]—and signals the need to anticipate the needs of an aged population. Further, the National Development Plan 2030 that aims to increase life expectancy of South Africans from 61 to 70 years, propagates for integrated health care delivery through the life course [45]. The life-course approach increases the effectiveness of interventions by targeting the needs of individuals at critical points in their lives [46]. In South Africa, screening older adults for multimorbidity will enable the identification of individuals needing treatment.

Our study found a positive association between multimorbidity and the highest wealth quintile. Studies on the African continent (e.g., Burkino Faso [47], Ghana [17], South Africa [48]) have shown that wealthiest groups had higher levels multimorbidity compared to the less wealthy groups. This contrasts with what has been noted in high income countries. In LMICs, this could be explained by wealthier people having access to high-calorie foods, tobacco, alcohol and other factors that can increase the risk of developing multiple conditions [18]. It is possible that residual confounding existed in our regression model and we were not able to fully separate the effects of wealth on education, occupation and BMI. Of course, the relationship with wealth may be mediated or confounded by education and occupation. Wealth and prosperity tends to be related to higher BMI in South Africa [49, 50], a factor also associated with multimorbidity. Obesity risk in African population groups may also be influenced by cultural norms that associate fatness with beauty [51].

Access to healthcare may also contribute to explain the association between multimorbidity and wealth. Since South Africa has both private and public health sector systems [52], wealthier people tend to have better access to healthcare in terms of private health insurance or medical aid. For example, many private medical aid schemes in the country offer—or even require mandatory—annual health screening benefits (blood pressure, BMI, glucose and cholesterol), which would likely increase awareness of these health conditions among those on higher wealth quintiles.

The common disease classes identified among the multimorbid population were “Diabetes and Hypertension,” “Heart disease and Hypertension” and “Stroke and Hypertension.” The largest disease class in our study was “Diabetes and Hypertension.” This combination of diseases has been previously identified in the literature both regionally [53–56] and internationally [57]. A multi-country study based in sub-Saharan Africa found that hypertension was the most common co-morbidity in a cohort of diabetes patients and was present in approximately 71% of patients with diabetes [58]. An analysis of a South African national HIV survey also identified “Diabetes and Hypertension” and “Heart disease and Hypertension” as disease classes in the multimorbid population [59]. Hypertension is a condition in which blood vessels have persistently raised pressure and if left untreated, can cause chest pain (angina), heart attacks, heart failure, and an irregular heartbeat, which can lead to a sudden death [60]. It can also cause strokes by blocking or bursting arteries that supply blood or oxygen to the brain [60]. Hypertension has many short and long term consequences [61]—such as the disease conditions identified as co- or multi-morbid in this study (diabetes, stroke, heart disease or heart failure). The number of people with hypertension is increasing and detection but treatment rates remain strikingly low (control rates are below 13% in Sub-Saharan Africa) [62]. While prevention and screening are important, of equal importance is the need to effectively manage patients. To reduce fragmentation of care and meet the needs of people with multimorbidity, several European countries have introduced disease management programmes focused on integrated care [63]. The World Health Organization has also suggested that integrated care is beneficial for older people [64]. Given the growing burden of NCDs in many LMICs, integrated care aimed at reducing and managing the burden of NCDs need to be investigated.

### Limitations

This study was a secondary analysis of survey data and was limited to the data reported in the survey. Since the NIDS 2017 survey collected information on few disease conditions, this analysis was also limited in the number of disease conditions included. We used a combination of measured and self-reported health data. The self-reported disease condition data was most likely underestimated which would, in turn, lead to an underestimation in the prevalence of multimorbidity. We also included a few disease conditions which may also lead to an underestimation in the assessed prevalence of multimorbidity. In addition, we assumed that missing data for a disease condition meant no disease was present which may have led to a lower estimation of prevalence for disease conditions. The prevalence of multimorbidity in this study was lower compared to other local studies [53]. However, some multimorbidity prevalence studies that included younger people in South Africa also found lower prevalences i.e. between 6 and 13% [59, 65, 66]. The prevalence in this study was very similar to the prevalence estimates observed in another study of the 2008 NIDS Wave 1 (4.0%) [48] and the 2010 NIDS Wave 3 (2.8%) [67]. This could be due to the NIDS being a panel survey that goes back to interview a similar panel of participants. It is also most likely due to the underestimated prevalence of self-reported single diseases found in all three studies based on the NIDS surveys. While each study included variations of disease conditions, all three studies included hypertension and diabetes.

The estimated hypertension prevalence in this analysis appears to be plausible. For example, in this study, approximately 28% of the population 15 years and older was found to have hypertension (27.3% in males and 28.4% in females). In comparison, a meta-regression of hypertension estimates for the population 25 years and older was found to be 38.9% for males and 40% for females [68]. However, the diabetes prevalence in this study was 2.9% whereas other national surveys of people aged 15 years and older (using biomarkers) have placed the diabetes prevalence closer to 14.7% in 2012 [69] and 14.9% in 2016 [29]. A meta-regression of diabetes prevalence in people 25 years and older determined the prevalence to be 12.8% [70]. This indicates that the diabetes prevalence was most likely underestimated in the 2017 NIDS Wave 5, probably because it was self-reported. In a comparison of self-reported diabetes prevalence and prevalence based on HbA1c in another survey, the author concluded that 61% of cases of diabetes were likely to be undiagnosed [71]. As “Diabetes and Hypertension” make up the largest disease class in this study, any underestimation in the prevalence of these two diseases would have impacted the estimation of multimorbidity prevalence. While multimorbidity prevalence may be underestimated in our study, we still had important findings on the factors associated with multimorbidity and disease patterns.

### Conclusion

This study builds upon previous studies that examined multimorbidity in earlier waves of the NIDS dataset. As in the previous studies, the multimorbidity prevalence remained low but this is most likely due to the under-reporting of disease conditions. There was still a substantial amount of morbidity in the population, especially due to hypertension which reached extremely high levels among older people. Our study highlighted that age, belonging to the highest wealth quintile, current smoking, being overweight or obese increased the odds of multimorbidity; whereas having secondary education was protective of multimorbidity. Slightly more than half of the participants with multimorbidity were estimated to belong to the “Diabetes and Hypertension” class. Integrated models of care are needed to prevent, manage and optimise the treatment of NCD multimorbidity.

## Data Availability

The NIDS 2017 data is available on the DataFirst website (https://www.datafirst.uct.ac.za/dataportal/index.php/catalog/712).
