# Supplementary material for: Inequity in the Distribution of Non-Communicable Disease Multimorbidity in Adults in South Africa: An Analysis of Prevalence and Patterns
Source: Int J Public Health. 2022 Aug 16;67:1605072. doi: 10.3389/ijph.2022.1605072 (PMC9426027; doi:10.3389/ijph.2022.1605072)
Supplement: Supplementary file 1 [file DataSheet1.docx]

**Supplementary material**

**Table S1. Self-reported disease questions from questionnaire**

| Disease condition | Question |
| --- | --- |
| Diabetes or blood sugar | Have you ever been told by a doctor, nurse or health care professional that you have Diabetes or high blood sugar? |
| Heart disease | Have you ever been told by a doctor, nurse or health care professional that you have heart problems? |
| High blood pressure | Have you ever been told by a doctor, nurse or health care professional that you have High Blood Pressure |
| Stroke | Have you ever been told by a doctor, nurse or health care professional that you have Stroke? |

**Table S2. Description of missing data (South Africa, 2017, unweighted)**

| Variable | Total (%, n)  *N= 27 042* | Missing values  (%, n) |
| --- | --- | --- |
|  |  |  |
| Age (Median and interquartile range in years)^ | 33 (23 -51) | 0.07 (20) |
| Sex |  |  |
| Male | 43.2 (11 659) | 008 (21) |
| Female | 56.9 (15 362) |  |
| Locality |  |  |
| Rural | 44.4 (11 992) | 0 (0) |
| Urban | 55.7 (15 050) |  |
| Province |  |  |
| Western Cape | 11.5 (3 099) | 0 (0) |
| Eastern Cape | 11.1 (3 012) |  |
| Northern Cape | 7.2 (1 936) |  |
| Free State | 5.5 (1 493) |  |
| KwaZulu-Natal | 28.6 (7 740) |  |
| North West | 6.1 (1 640) |  |
| Gauteng | 14.6 (3 960) |  |
| Mpumalanga | 7.2 (1 954) |  |
| Limpopo | 8.2 (2 208) |  |
| Education level |  |  |
| Primary or less | 23.6 (6 320) | 0.81 (219) |
| Secondary complete | 63.2 (16 952) |  |
| Tertiary | 13.2 (3 551) |  |
| Employed | 33.9 (9 157) | 0 (0) |
| Individual Income (Mean & Standard Deviation ZAR) ^~^ | 2306.6 (6970) | 0 (0) |
| Private health insurance | 11.3 (26 90) | 11.8 (3 199) |
| Body Mass Index |  |  |
| Underweight | 8.0 (1 870) | 13.9 (3 746) |
| Normal weight | 42.2 (9 821) |  |
| Overweight | 22.8 (5 306) |  |
| Obesity grade 1 | 14.6 (3 397) |  |
| Obesity grade 2 | 7.4 (1 733) |  |
| Obesity grade 3 | 5.0 (1 169) |  |
| Current smoker | 17.7 (4 225) | 11.93 (3 226) |
| Hypertension | 29.52 (6 951) | 12.93 (3 497) |
| Diabetes | 3.89 (981) | 6.65 (1 799) |
| Heart disease | 1.74 (442) | 6.01 (1 625) |
| Stroke | 1.03 (263) | 5.46 (1 477) |

**Table S3. Description of sample by sex (South Africa, 2017, unweighted)**

| Variable | % (n) | | | P-value* |
| --- | --- | --- | --- | --- |
|  | Total  (N= 27 042) | Male  (n= 11 659) | Female  (n= 15 362) |  |
| Age  (Median years and IQR) | 33 (23 -51) | 31 (22 – 47) | 35 (24 – 53) | **<0.001** |
| Urban location | 55.7 (15 050) | 58.2 (6 793) | 53.7 (8 255) | **<0.001** |
| Province |  |  |  | **<0.001** |
| Western Cape | 11.5 (3 099) | 12.1 (1 409) | 11.0 (1 689) |  |
| Eastern Cape | 11.1 (3 012) | 10.8 (1 257) | 11.4 (1 754) |  |
| Northern Cape | 7.2 (1 936) | 7.7 (893) | 6.8 (1 043) |  |
| Free State | 5.5 (1 493) | 5.6 (655) | 5.5 (837) |  |
| KwaZulu-Natal | 28.6 (7 740) | 26.7 (3 111) | 30.1 (4 621) |  |
| North West | 6.1 (1 640) | 6.3 (729) | 5.9 (909) |  |
| Gauteng | 14.6 (3 960) | 15.8 (1 844) | 13.7 (2 110) |  |
| Mpumalanga | 7.2 (1 954) | 7.5 (871) | 7.1 (1 083) |  |
| Limpopo | 8.2 (2 208) | 7.6 (890) | 8.6 (1 316) |  |
| Education level |  |  |  | **<0.001** |
| Primary or less | 23.6 (6 320) | 21.0 (2 418) | 25.5 (3 899) |  |
| Secondary complete | 63.2 (16 952) | 66.3 (7 644) | 60.9 (9 294) |  |
| Tertiary | 13.2 (3 551) | 12.8 (1 471) | 13.6 (2 076) |  |
| Employed | 33.9 (9 157) | 38.9 (4 533) | 30.1 (4 619) | **<0.001** |
| Individual Income^~^ (Mean and standard deviation in ZAR) | 2306.6 (6970) | 3267.5 (8631.4) | 1576.0 (5260.3) | **<0.001** |
| Asset index |  |  |  | **<0.001** |
| Quintile 1 (Poorest) | 21. 2 (4 772) | 19.7 (1926) | 22.3 (2842) |  |
| Quintile 2 | 19.9 (4 496) | 20.0 (1959) | 19.9 (2534) |  |
| Quintile 3 | 19.8 (4 470) | 19.8 (1936) | 19.9 (2531) |  |
| Quintile 4 | 20.1 (4 541) | 21.0 (2050) | 19.6 (2491) |  |
| Quintile 5 (Richest) | 18.9 (4 264) | 19.6 (1914) | 18.4 (2343) |  |
| Private health insurance | 11.3 (2 690) | 12.3 (1200) | 10.6 (1487) | **<0.001** |
| BMI |  |  |  | **<0.001** |
| Underweight | 8.0 (1 870) | 13.2 (1 258) | 4.4 (612) |  |
| Normal weight | 42.2 (9 821) | 57.2 (5 450) | 31.7 (4 371) |  |
| Overweight | 22.8 (5 306) | 19.4 (1 846) | 25.1 (3 460) |  |
| Obesity grade 1 | 14.6 (3 397) | 7.4 (700) | 19.6 (2 697) |  |
| Obesity grade 2 | 7.4 (1 733) | 2.1 (199) | 11.1 (1 534) |  |
| Obesity grade 3 | 5.0 (1 169) | 0.7 (68) | 8.0 (1 101) |  |
| Current smoker | 17.7 (4 225) | 32.5 (3 158) | 7.6 (1 064) | **<0.001** |

Note: There were 21 observations with missing sex information. *Chi-square tests used, and Wilcoxon rank-sum test used for Age and Individual income variables. ^~^Median income was R0.

**Table S4. Prevalence of single disease conditions by wealth quintiles (South Africa, 2017)**

| Disease conditions | Weighted disease prevalence (%, 95%CI) | | | | | |
| --- | --- | --- | --- | --- | --- | --- |
|  | **Total** | **Wealth quintiles** | | | | |
|  |  | **Q1 / Least wealthy** | **Q2** | **Q3** | **Q4** | **Q5 / Most wealthy** |
| Diabetes | 2.9  (2.6-3.3) | 1.9  (1.4-2.5) | 2.3  (1.8-3.0) | 2.1  (1.6-2.8) | 2.6  (2.0-3.4) | 4.6  (3.8-5.6) |
| Heart disease | 1.6  (1.3-1.8) | 1.0  (0.7-1.5) | 1.0  (0.6-1.6) | 1.0  (0.8-1.4) | 1.6  (1.1-2.3) | 2.5  (1.8-3.3) |
| Hypertension | 27.8  (26.7-29.0) | 22.5  (20.5-24.6) | 24.8  (22.6-27.1) | 25.6  (23.4-28.0) | 29.7  (27.3-32.3) | 34.3  (31.8-36.9) |
| Stroke | 0.8  (0.6-1.0) | 0.7  (0.5-1.2) | 0.9  (0.6-1.3) | 0.5  (0.3-0.9) | 0.6  (0.3-1.0) | 1.0  (0.7-1.5) |

**Table S5. Prevalence of disease conditions by sex (South Africa, 2017, unweighted and weighted percentages)**

| Disease conditions | Unweighted prevalence by sex (%, n/N) | | | |
| --- | --- | --- | --- | --- |
|  | **Total**  **(n= 27 042)** | **Male**  **(n= 11 659)** | **Female**  **(n= 15 362)** | ***P-*value** |
| Diabetes | 3.9 (981) | 2.9 (316) | 4.6 (664) | **<0.001** |
| Heart disease | 1.7 (442) | 1.2 (132) | 2.1 (309) | **<0.001** |
| Hypertension | 29.5 (6 951) | 27.0 (2 591) | 31.3 (4 358) | **<0.001** |
| Stroke | 1.0 (263) | 1.0 (110) | 1.0 (153) | 0.838 |
| **Disease conditions** | **Weighted prevalence by sex (%, 95% CI)** | | | |
|  | **Total** | **Male** | **Female** | **-** |
| Diabetes | 2.9 (2.6-3.3) | 2.1 (1.7-2.5) | 3.3 (3.8-4.3) | **-** |
| Heart disease | 1.6 (1.3-1.8) | 1.2 (0.9-1.5) | 1.9 (1.6-2.3) | **-** |
| Hypertension | 27.8 (26.7-29.0) | 27.3 (25.6-29.0) | 28.4 (27.1-29.7) | **-** |
| Stroke | 0.8 (0.6-1.0) | 0.7 (0.5-0.9) | 0.9 (0.7-1.1) | **-** |

|  |
| --- |
|  |
|  |

**Fig S1. Disease prevalence by age group in A) males and B) females; and C) multimorbidity disease prevalence in persons by age group. Note that all graphs are weighted. (South Africa, 2017)**

**Table S6. Number of diseases in individuals by sex (South Africa, 2017, unweighted and weighted).**

| Number of diseases | Unweighted prevalence of disease by sex (%, n/N) | | | |
| --- | --- | --- | --- | --- |
|  | **Total**  **(n= 27 042)** | **Male**  **(n= 11 659)** | **Female**  **(n= 15 362)** | ***P-*value** |
| No diseases | 72.2 (19 516) | 75.7 (8 830) | 69.5 (10 669) | **<0.001** |
| 1 disease | 24.2 (6 555) | 21.9 (2 550) | 26.0 (4 001) | **<0.001** |
| 2 diseases | 3.1 (835) | 2.1 (241) | 3.9 (594) | **<0.001** |
| 3+ diseases | 0.5 (136) | 0.3 (38) | 0.6 (98) | **<0.001** |
| **Multimorbidity**  **(≥ 2 diseases)** | **3.6 (971)** | **2.4 (279)** | **4.5 (692)** | **<0.001** |
| **Number of diseases** | **Weighted prevalence of disease by sex (%, 95% CI)** | | | |
|  | **Total** | **Male** | **Female** | **-** |
| No diseases | 74.5 (73.5-75.4) | 76.0 (74.5-77.5) | 71.7 (73.0-74.2) | **-** |
| 1 disease | 22.8 (21.8-23.8) | 22.1 (20.7-23.6) | 22.3 (23.4-24.6) | **-** |
| 2 diseases | 2.3 (2.1-2.6) | 1.5 (1.2-1.9) | 2.7 (3.1-3.5) | **-** |
| 3+ diseases | 0.4 (0.3-0.5) | 0.3 (0.2-0.5) | 0.4 (0.5-0.7) | **-** |
| **Multimorbidity**  **(≥ 2 diseases)** | **2.7 (2.4-3.1)** | **1.8 (1.5-2.3)** | **3.6 (3.2-4.0)** | **-** |

**Table S7. Prevalence of disease in multimorbid population by sex (South Africa, 2017, weighted)**

| Disease condition | Persons | Males | Females |
| --- | --- | --- | --- |
|  | **% (95% CI)** | | |
| Hypertension | 98.9 (97.6-99.5) | 98.1 (95.9-99.2) | 99.2 (97.0-99.8) |
| Diabetes | 68.2 (63.0-72.9) | 68.7 (59.1-76.9) | 67.9 (62.0-73.3) |
| Heart disease | 37.5 (32.4-43.0) | 35.3 (26.0-46.0) | 38.6 (32.9-44.6) |
| Stroke | 16.1 (13.2-19.4) | 20.1 (14.4-27.4) | 14.1 (11.0-18.0) |

**Table S8. Fit statistics for latent class analysis models with different numbers of classes (South Africa, 2017)**

| Fit statistics | Latent class analysis models | | | |
| --- | --- | --- | --- | --- |
|  | **2 classes** | **3 classes** | **4 classes** | **5 classes** |
| Design effect | 1.5 | 1.2 | - | - |
| DF | 6 | 1 | -4 | -9 |
| Entropy R-sqd | 1.0 | 1.0 | 0.9 | 0.9 |
| Entropy Raw | 32.1 | 49.3 | 100.9 | 119.9 |
| Adjusted BIC | 265.8 | 82.5 | 85.4 | 102.2 |
| BIC | 294.4 | 127.0 | 145.8 | 178.4 |
| AIC | 250.5 | 58.7 | 53.1 | 61.3 |
| G-squared | 232.5 | 30.7 | 15.1 | 13.3 |
| Log Likelihood | -1459.2 | -1358.3 | -1350.5 | -1349.6 |

**Table S9. Membership probability and standard errors (South Africa, 2017, weighted)**

| Disease class | Membership probability (SE) |
| --- | --- |
| Stroke and Hypertension | 0.153 (0.019) |
| Heart disease and Hypertension | 0.321 (0.026) |
| Diabetes and Hypertension | 0.526 (0.030) |
